# Cancer-Specific Survival of Patients with Non-Muscle-Invasive Bladder Cancer: A Population-Based Analysis

**DOI:** 10.1245/s10434-023-14051-9

**Published:** 2023-08-14

**Authors:** Aleksander Ślusarczyk, Piotr Zapała, Łukasz Zapała, Tomasz Borkowski, Piotr Radziszewski

**Affiliations:** Department of General, Oncological, and Functional Urology, Medical University of Warsaw, Warsaw, Poland

**Keywords:** Non-muscle-invasive bladder cancer, Nomogram, Cancer-specific survival, Stage, Age

## Abstract

**Background and Purpose:**

Non-muscle-invasive bladder cancer (NMIBC) constitutes a heterogeneous group of tumors with different prognoses. This population-based study aimed to report real-world cancer-specific survival (CSS) of NMIBC and create a prognostic nomogram based on the identified risk factors.

**Methods:**

The Surveillance, Epidemiology, and End Results database was searched for patients diagnosed with NMIBC from 2004 to 2015, who underwent transurethral resection of the bladder tumor. The dataset was divided into development and validation cohorts. Factors associated with CSS were identified using Cox proportional hazards and used to develop a prognostic nomogram.

**Results:**

In total, 98,238 patients with NMIBC were included. At the median follow-up of 124 months (IQR 81–157 months), cancer-specific mortality (CSM) was highest for T1HG (19.52%), followed by Tis (15.56%), similar for T1LG and TaHG (10.88% and 9.23%, respectively), and lowest for TaLG (3.76%). Multivariable Cox regression for CSS prediction was utilized to develop a nomogram including the following risk factors: tumor T category and grade, age, tumor size and location, histology type, primary character, race, income, and marital status. In the validation cohort, the model was characterized by an AUC of 0.824 and C-index that reached 0.795.

**Conclusions:**

To conclude, NMIBC is associated with a significant risk of long-term CSM especially, but not only, in patients with T1HG. Rarely diagnosed TaHG and T1LG tumors should be regarded as high-risk due to approximately 10% CSM. T category, grading, and age remain the most powerful determinants of CSS in NMIBC, but sociodemographic factors might also influence its prognosis.

**Supplementary Information:**

The online version contains supplementary material available at 10.1245/s10434-023-14051-9.

The majority of patients with bladder malignancy are diagnosed with non-muscle-invasive bladder cancer (NMIBC). NMIBC comprises non-invasive papillary tumors (Ta), carcinoma in situ (Tis) and tumors invading the submucosa (T1). NMIBCs are treated surgically by transurethral resection of the tumor (TURBT), followed by adjuvant intravesical Bacillus Calmette-Guérin (BCG) or chemotherapy in intermediate- and high-risk patients. Major risk factors for the recurrence and progression of NMIBC are grade, tumor T category, concurrent Tis, prior recurrence rate, multiplicity, and tumor diameter.^1,2^ Grading and tumor T category remain the strongest risk factors for NMIBC progression.^3^ High-grade (HG) T1 tumors are the most aggressive among all NMIBCs with 5-year cancer-specific mortality (CSM) of approximately 10%.^3^ Conversely, low-grade (LG) Ta tumors are characterized by good prognosis and rarely progress.^4^

Outcomes of rarely diagnosed TaHG and T1LG are not as well-evaluated, due to the low frequency of these stages. TaHG tumors are rare and constitute around 4% of all papillary TaT1 tumors.^5^ The existence of low-grade T1 is sometimes questioned as the invasive ability of the cancer is related to the presence of malignant undifferentiated cells that infiltrate the submucosa, which implies that T1 cannot be low-grade. However, a study by van de Putte confirmed the presence of low-grade/grade 2 T1, whereas the study by Beijert et al. reported the existence of T1 grade 1 tumors.^6,7^

The vast majority of prognostic models and nomograms for NMIBC stratify patients according to the risk of recurrence and progression, which constitute the most frequently met endpoints.^1–3^ Cancer-specific survival (CSS) of patients with NMIBC is usually not investigated, as they relatively rarely die of bladder cancer (BC).^3,8^ Only large-population or multicenter studies can provide reliable data for the assessment of CSS prognostic factors in NMIBC. We lack real-world robust data on the survival outcomes and prognostic factors in NMIBC.

In this population-based study, we aimed to report real-world cancer-specific mortality of different NMIBC stages and develop a prognostic nomogram for CSS in NMIBC patients.

## Methods

The National Cancer Institute Surveillance, Epidemiology, and End Results (SEER) database, based on 17 different registries, was used for this research. A systematic search was performed to identify patients with NMIBC according to the 6th Derived American Joint Committee on Cancer (AJCC) T edition (ICD codes from C67.0 to C67.9 with the exclusion of C67.7). In the subsequent selection, only patients treated with TURBT between 2004 and 2015 were included in the analysis. Exclusion criteria were as follows: any evidence of lymph node involvement or distant metastases, previous history of MIBC, lack of histopathological confirmation of BC, discrepancy in reporting tumor stage and grade, previous radiotherapy for BC, missing tumor grading or T category, unknown survival status.

The available data included information on the patient’s demographics, tumor histopathological (e.g., T category, grading) and clinical characteristics (e.g., tumor size, location, history of previous recurrence), therapy used (e.g., surgery type, chemotherapy), and survival outcomes (cancer-specific and all-cause mortality during the follow-up).

### Ethics

The institutional review board waived the need for study approval. The study was performed in accordance with the Declaration of Helsinki and its later amendments.

### Statistical Analysis

The dataset was divided into development and validation cohorts in the proportion of 70 to 30. Group characteristics are presented as the number of patients and percentage for categorical variables. Median survival follow-up was computed using the reverse Kaplan-Meier method.

Cox proportional hazard regression was performed to identify factors prognostic for CSS and overall survival (OS). Independent risk factors used in the multivariable analysis were used to create a prognostic nomogram for 5-year CSS. A hazard ratio (HR) supplemented with a 95% confidence interval (95% CI) was derived. For all analyses, we considered a two-sided *p* value <  0.05 as statistically significant. Calibration plots were generated to illustrate the nomogram-predicted and actual outcomes. The concordance index (C-index) was calculated for development and validation cohorts to demonstrate the discrimination of the nomogram. The area under the receiver operating characteristic curve (AUC) was calculated to measure the accuracy. Furthermore, decision curve analysis (DCA) was performed to illustrate the net benefit for the nomogram. Statistical analyses were performed using SAS software version 9.4 (Cary, North Carolina, U.S.) and R studio using R programming language version 4.2.1 (R Foundation for Statistical Computing, Vienna, Austria) with ‘rms’, ‘gtsummary,’ and ‘dcurves’ packages.

## Results

### Cohort Characteristics

Overall, 98,238 patients with NMIBC who underwent TURBT were included in the analysis. Patients were diagnosed with the following tumor stages: 24,466 (24.9%) patients with high-grade T1, 7718 (7.86%) with low-grade T1, 16,365 (16.66%) with high-grade Ta, 47,671 (48.53%) with low-grade Ta, and 2018 (2.05%) with carcinoma in situ. The most numerous group consisted of patients who were between 70 and 80 years old (30.71%), followed by 25.53% and 26.08% of patients between 60 and 70 years and over 80 years of age, respectively; whereas 17.69% of patients were younger than 60 years old. Males constituted 76.89% of the cohort. Details on baseline patient characteristics are summarized in Table 1.

**Table 1:** Baseline characteristics of the whole cohort of patients with non-muscle-invasive bladder cancer

Characteristics		Whole cohort	
		Number of patients	%
Gender	Male	75,534	76.89
	Female	22,704	23.11
Age (years)	<60	17,383	17.69
	60-70	25,076	25.53
	70-80	30,166	30.71
	>80	25,613	26.08
Tumor category and grading	T1HG	24,466	24.90
	T1LG	7718	7.86
	TaHG	16,365	16.66
	TaLG	47,671	48.53
	Tis	2018	2.05
Tumor histology	Urothelial	97,059	98.80
	Squamous	700	0.71
	Other	479	0.49
Tumor location	Lateral/posterior/trigone	44,117	44.91
	Anterior/dome	4848	4.93
	Bladder neck	2920	2.97
	More than one area	8856	9.01
	Not specified/ multiple	37,497	38.17
Primary tumor	Recurrent	1059	1.08
	Primary	97,179	98.92
Tumor size	< 3 cm	22,514	22.92
	≥ 3 cm	23,621	24.04
	Unknown	52,103	53.04
Race	White	88,859	90.45
	Black	4376	4.45
	Other*	5003	5.09
Marital status	Unmarried	31,027	31.58
	Married **	60,870	61.96
	Status unkown	6341	1.06
Income annually	< $65,000	43,580	44.36
	≥ $65,000	54,652	55.64
Metropolitan citizenship***	No	13,168	13.41
	Yes	85,017	86.59
Survival (months)	Median/IQR	124	81-157
Cancer-specific death	No	89,006	90.60
	Yes	9232	9.4
Death	No	50,620	51.53
	Yes	47,618	48.47

*Includes American Indian/Alaska Native/Asian or Pacific Islander.

**Partnership without official marriage was also regarded as married status.

***Citizenship of counties in a metropolitan area.

### Cancer-Specific Mortality

At the median follow-up of 124 months (IQR 81–157 months), CSM was highest for T1HG (19.52%), followed by high-grade Tis (15.56%), similar for T1LG and TaHG (10.88% and 9.23%, respectively), and lowest for TaLG tumors (3.76%). For the whole cohort of NMIBCs, 5- and 10-year CSS were 89.6% and 83.3%, respectively.

### Factors Predicting Cancer-Specific Survival

**Fig. 1:**
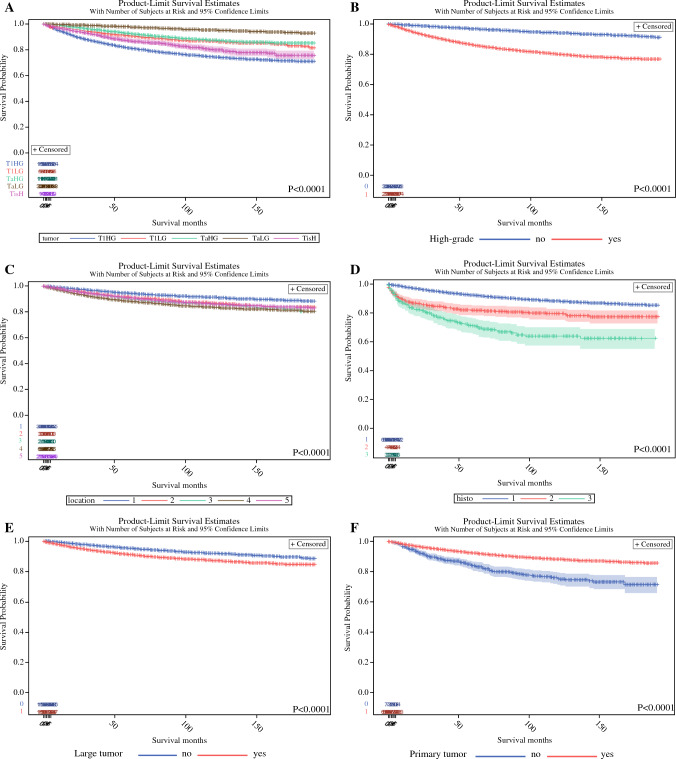
The association between the tumour stage (**A**), grade (**B**), location (**C**), histology (**D**), size (**E**), primary character (**F**), and cancer-specific survival of NMIBC patients. Legend: location (*1*- lateral/ posterior/ trigone; 2- anterior/dome; 3- neck; 4- more than one area; 5- not specified/multiple); histology (1- urothelial/2- squamous/3- other types).

**Fig. 2.**
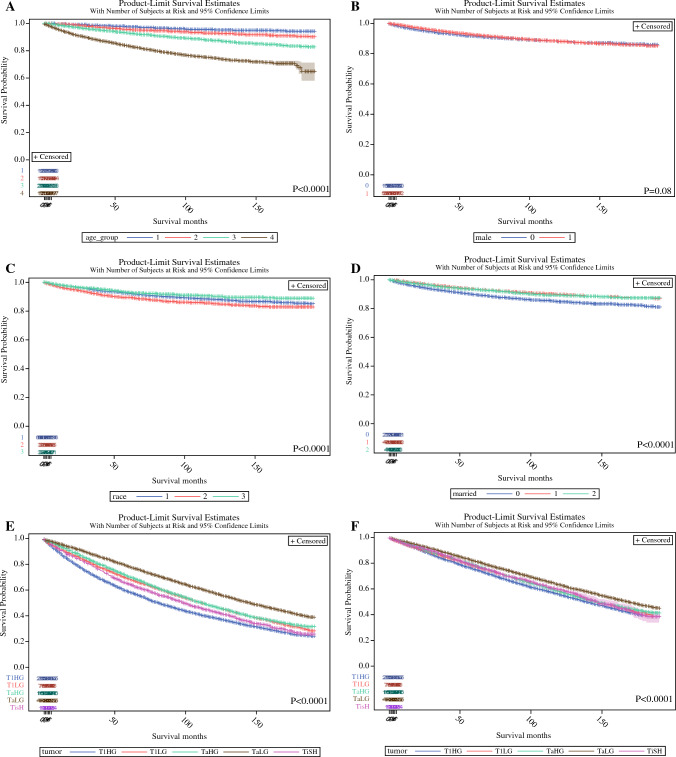
The association between age (**A**), gender (**B**), race (**C**), marital status (**D**), and cancer-specific survival of NMIBC patients. The association between tumour stage with overall survival (**E**), and other-cause mortality (**F**). Legend: age (1- < 60 yr; 2- 60-70 yr; 3- 70-80 yr; 4- >80 years); male (no/yes); race (1-White /2- Black/3- other); 3- married (0- no/1- yes/2- unknown).

The Kaplan-Meier analyses demonstrated several prognostic factors influencing CSS (Figs. 1A–J and 2A–D). Tumor T category and grade stratified patients according to CSS. Patients with T1HG tumors had the worst CSS followed by patients with high-grade Tis, T1LG and TaHG, and TaLG. Patients with larger tumor size, recurrent bladder tumor, with tumor located in the bladder neck or multiple/non-specified tumor sites had worse CSS. Male gender and Black race were also associated with unfavorable CSS.

**Fig. 3:**
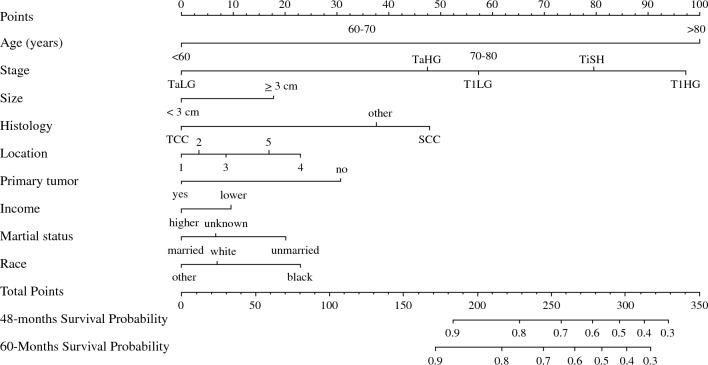
Prognostic nomogram for cancer-specific survival in non-muscle-invasive bladder cancer. Legend: Age groups (years), Bladder tumor location: 1- trigone or posterior or lateral walls; 2- anterior wall or dome; 3- bladder neck; 4- tumor in overlapping sites; 5- non-otherwise specified location, including multiple tumors.

Multivariable analysis with Cox proportional hazards regression identified factors associated with CSS (Table 2). Tumor stage (for T1HG HR = 5.38 95% CI 4.85–5.98; for Tis HR = 4.29 95% CI 3.37–5.47; for T1LG HR = 2.53 95% CI 2.16–2.96; for TaHG HR = 2.40 95% CI 2.12–2.74; compared with TaLG HR = 1.0; *p* < 0.001;), patient’s age (for 60–70 years HR = 1.55 95% CI 1.33–1.82; for 70–80 years HR = 2.41 95% CI 2.08–2.80; for > 80 years HR = 5.25 95% CI 4.54–6.07; compared with < 60 years HR = 1.0; *p* < 0.001), large tumor size (HR = 1.31 95% CI 1.21–1.42; *p* < 0.001; ≥ 3 cm vs. < 3 cm), histology type (HR = 1.32 95% CI 1.11–1.55; *p* < 0.001; squamous cell vs urothelial), tumor location (for tumor of overlapping sites HR = 1.44 95% CI 1.28–1.63; for not specified/multiple tumors HR = 1.32 95% CI 1.21–1.44; compared with lateral/posterior/trigone HR = 1.0 *p* < 0.001), recurrent tumor (HR = 1.91 95% CI 1.40–2.62; *p* < 0.001; recurrent vs. primary), patient’s race (HR = 1.32 95% CI 1.11–1.55; *p* < 0.001; Black vs White), income (HR = 1.19 95% CI 1.10–1.28; *p* < 0.001; lower vs higher) and marital status (HR = 1.36 95% CI 1.25–1.47; *p* < 0.001; unmarried vs married) constituted independent risk factors for CSS (Table 2). The above factors derived from the multivariable analysis were used to create a nomogram prognostic for 5-year CSS (Fig. 3).

**Table 2:** Multivariable analysis with Cox proportional hazards for the prediction of cancer-specific survival of patients with non-muscle-invasive bladder cancer

Factors predicting cancer-specific survival—multivariable analysis				
Variables		HR	95% CI	*P*-value
Tumor T category and grading	TaLG	ref		<.0001
	T1LG	2.532	2.163–2.963	<.0001
	TaHG	2.409	2.116–2.743	<.0001
	Tis	4.290	3.367–5.465	<.0001
	T1HG	5.382	4.847–5.976	<.0001
Age (years)	<60	ref		0.0001
	60-70	1.552	1.325–1.818	<.0001
	70-80	2.411	2.078–2.797	<.0001
	>80	5.252	4.541–6.074	<.0001
Tumor size	≥ 3 cm vs < 3 cm	1.308	1.206–1.419	<.0001
Tumor histology	Urothelial	ref		<.0001
	Squamous	1.315	1.112–1.554	<.0001
	Other	0.861	0.718–1.033	0.0001
Tumor location	Lateral/ posterior/ trigone	ref		<.0001
	Anterior/dome	1.087	0.926–1.276	0.3058
	Bladder neck	1.141	0.907–1.436	0.2611
	More than one area	1.445	1.281–1.630	<.0001
	Not specified/multiple	1.319	1.207–1.441	<.0001
History of previous NMIBC	Yes vs no	1.911	1.397–2.615	<.0001
Income	< $65,000 vs ≥ $65,000	1.186	1.097–1.281	<.0001
Marital status	Married*	ref		<.0001
	Unmarried	1.359	1.254–1.473	<.0001
	Status unkown	1.084	0.892–1.316	0.4166
Race	White	ref		0.0012
	Black	1.315	1.112–1.554	0.0013
	Other**	0.861	0.718–1.033	0.1064

*Partnership without official marriage was also regarded as married status.

**Includes American Indian/Alaska Native/Asian or Pacific Islander.

### Overall and Other-Cause-Specific Survival

Estimated 5-year and 10-year OS reached 71.3% and 50.3%, respectively. Tumor stage was significantly associated with OS (Fig. 2E) and other-cause-specific survival (Fig. 2F). Other-cause-specific survival was the worst in T1HG and the most favorable in TaLG patients (Fig. 2F). Overall, 47,618 all-cause deaths were recorded within the median follow-up of 124 months. The main mortality causes included cardiovascular diseases (N = 12,977; 27.3%), bladder cancer (N = 9232; 19.4%), respiratory diseases (N = 4052; 8.5%), lung cancer (N = 3042; 6.4%), dementia (N = 1337; 2.8%), diabetes mellitus (N = 1040; 2.2%), prostate cancer (N = 958; 2%), hematological malignancies (N = 897; 1.9%), infections (N = 827; 1.7%), upper urinary tract urothelial cancer (N = 499; 1%) and other causes (including other malignancies; N = 12,757; 26.8%). Multivariable analysis with Cox proportional hazards revealed that tumor T category and grade, patient’s age and gender, tumor size and location, histology type, primary character, patient’s race, income, metropolitan citizenship, and marital status were all associated with OS (Supplementary Table 1).

### CSS Nomogram Validation

**Fig. 4:**
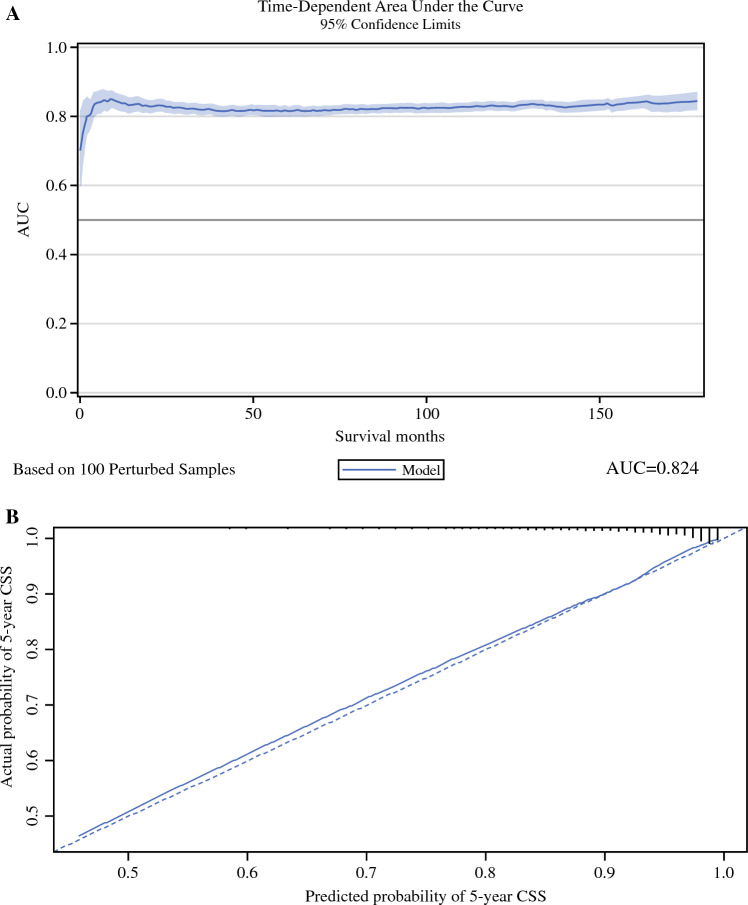
Time-dependent area under the curve (AUC) (**A**), and calibration plot (**B**) of the nomogram for the prediction of cancer-specific survival in the validation cohort

The CSS model was characterized by the time-dependent AUC (Fig. 4A) that reached 0.835 and 0.824 in the development and validation cohorts, respectively. The C-index of the nomogram achieved 0.787 and 0.795 in the development and validation cohorts, respectively.

**Fig. 5:**
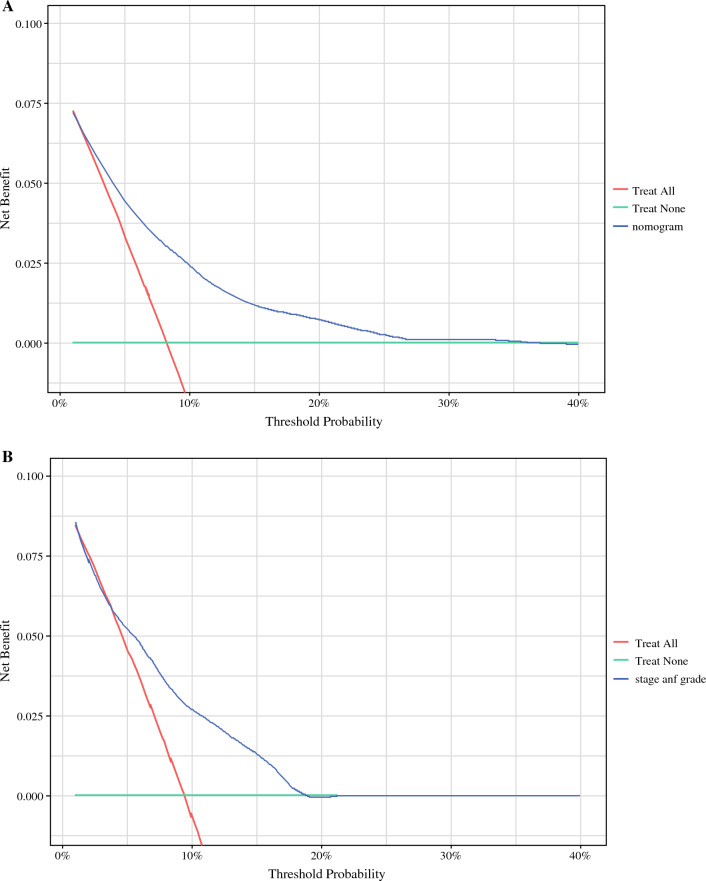
Decision curve analysis for the nomogram (**A**) and tumor stage and grade (Ta/T1/Tis/LG/HG) (**B**) in the prediction of 5-year cancer-specific survival in the validation cohort

The calibration plot and decision curve analysis for the nomogram are presented in Figs. 4 and 5, respectively. The nomogram was characterized by good calibration (Fig. 4B) and, as illustrated by the decision curve analysis (Fig. 5A), is clinically useful across the whole range of threshold probabilities. The net benefit was higher for the nomogram than for the model based solely on the tumor stage and grade (Tis/Ta/T1/LG/HG), especially at higher probability thresholds (Fig. 5B).

## Discussion

In this population-based study, we compared the prognosis of different NMIBC stages and developed a prognostic nomogram for CSS of NMIBC patients. Firstly, we identified tumor T category, histological grading, and patient’s age as the strongest predictors of CSS. We confirmed that among NMIBC, T1HG and Tis are characterized by the most unfavorable disease-specific survival (up to 19% of patients die due to BC). Rarely diagnosed T1LG and TaHG are also associated with a non-negligible risk of CSM (approximately 10%), whereas TaLG is associated with favorable survival. Secondly, we constructed a prognostic nomogram for 5-year CSS, which included tumor T category and grade, age, tumor size, histology type, tumor location, the primary character of the tumor, patient’s race, marital status, and socioeconomic status. Thirdly, internal validation of the nomogram confirmed its acceptably high accuracy, discrimination abilities, and good calibration. Fourthly, the clinical net benefit seems to be higher for our nomogram compared with conventionally used tumor T stage and grade in predicting 5-year CSS. Fifthly, the significant burden of other-cause-specific mortality among all stages of NMIBC emphasizes the importance of considering comorbidities during clinical decision-making.

Our data underscore the need for aggressive treatment for T1HG and Tis, but also careful management of “forgotten” and relatively rare TaHG and T1LG which are also deadly tumors (up to 10% CSM). T1HG is widely considered the most aggressive type of NMIBC with high progression rates and relatively poor survival prognosis (approximately 10% 5-year CSM). In our population-based study, T1HG was characterized by 81.5% CSS at the median follow-up of 124 months. This is rather consistent with the CSS reported in other studies summarized in the available systematic review (from 78% to 93% CSS).^9^ Because of such unfavorable outcomes, the debate on the indication for radical cystectomy (RC) in T1HG should be continued. In the treatment of NMIBC, RC has its established role in the BCG-unresponsive disease and tumors not amenable to complete transurethral resection. A recent systematic review of 10 studies demonstrated better 5- and 10-year CSS results for T1G3 patients who underwent early compared with deferred cystectomy.^10^ On the other hand, there is still no convincing evidence of the survival benefit from RC compared with conservative management with TURBT and adjuvant BCG in T1HG patients.^9^ The randomized controlled BRAVO-feasibility trial included a small cohort of patients who received either RC or a full course of BCG. None of the patients (0/20 patients) who underwent RC had developed metastatic progression at the study closure, compared with two patients with metastatic BC in the conservative management arm (2/25 patients).^11^ Results of large cohort randomized controlled trials are awaited, but accrual for such trials is poor. The prognosis of T1HG seems to improve over the years. In the historic, classical study, Shahin et al. suggested the rule of 30%; namely, 30% of patients never have a recurrence, 30% undergo deferred cystectomy and 30% die because of metastatic progression.^12,13^ On the other hand, competing risk of other-cause-specific mortality in patients with T1HG raises a concern about overtreatment when offering RC. As we demonstrated, T1HG patients have worse non-BC-specific survival than patients with TaLG. Perhaps, higher exposure to carcinogenic factors (e.g., smoking) leads to the development of more aggressive cancer, and simultaneously increases the risk of cardiovascular diseases and secondary malignancies contributing to non-BC mortality. The presence of more aggressive cancer in more comorbid patients poses a clinical dilemma as to the optimal therapy. Consequently, improved risk-stratification and taking comorbidities into consideration is obligatory to avoid over- and undertreatment in T1HG BC.

Our study indicates that TaHG is associated with nearly twice lower, but still substantial disease-specific mortality when compared to T1HG BC. A study by Quhal et al. showed that TaHG is associated with a substantially lower risk of progression than T1HG.^14^ Nevertheless, Bree et al. concluded that all TaHG tumors should be regarded as high-risk as they confer a significantly higher progression risk (approximately 6% TaHG progress to ≥ T2) than intermediate-risk low-grade Ta when treated with BCG.^15^ We believe that in light of this real-world data, TaHG should be always regarded as high-risk due to the substantial 9.2% CSM in the long-term observation.

Furthermore, T1LG is another rarely reported tumor category, as submucosa invasive tumors are suspected to consist of histologically undifferentiated cells able to infiltrate, which are classified as grade 3 according to WHO 1973 or high-grade according to WHO 2004 grading systems, respectively. A study by van de Putte et al., in which experienced pathologists re-revised T1 grading in 601 patients, yielded no grade 1 tumors but reported 188 (31%) grade 2 tumors and 413 (69%) grade 3 lesions according to the WHO 1973 classification.^6^ In the same study, according to the new WHO 2004 classification, 47 (6%) low-grade and 554 (94%) high-grade T1 tumors were diagnosed. In the aforementioned study, grade 3, but not high-grade constituted an independent prognostic factor for progression and CSS.^6^ Noteworthy, Beijert et al. performed a large multicenter retrospective study in which T1G1 was identified as rare (1.9% of all NMIBC), but with PFS similar to T1G2 and worse than TaG1.^7^ Available evidence and our real-world data suggest that despite their invasive character, T1 tumors might be low-grade, and this is independently associated with better prognosis. Nevertheless, T1LG should always be stratified into the high-risk group due to the significant risk of CSM.

Our nomogram underlines the crucial prognostic role of T category, grading, and age which are cardinal factors determining CSS. Moreover, we report the additional prognostic role of other tumor features and sociodemographic factors. So far, the EORTC 2016 nomogram has been the most commonly used for survival assessment of NMIBC. EORTC 2016 included T category and grade as risk factors for disease-specific survival; age and grade for OS.^3^ However, the above risk stratifications were validated only for intermediate- and high-risk patients treated with maintenance BCG therapy.^3^ Conversely, our nomogram is constructed for all NMIBCs and includes several more risk factors. Internal validation, decision-curve, and calibration analyses confirm its applicability in that setting. DCA confirms the advantage of our nomogram compared with conventional tumor T category and grade, which were proposed by Cambier et al. as risk factors in the EORTC 2016 nomogram.

We showed the impact of age not only on OS but also CSS. Age has been consistently reported as an adverse prognostic factor in other nomograms such as CUETO and EORTC 2016.^2,3^ Our study confirms the substantial role of age in BC prognosis, which might be not only related to a less aggressive therapeutic approach in elderlies, but also the biology of the tumor and impaired mechanisms of self-defense (e.g., immune surveillance) in older patients. Our previous study underscored the very high BC mortality and progression burden and suboptimal management in elderlies with T1HG BC.^16^

Factors of secondary relevance which, however, should also be considered, are: tumor size, tumor character (primary vs recurrent), histology type, and tumor location in the bladder. In general, histology types such as squamous cell, neuroendocrine, and micropapillary cancer are more aggressive than urothelial cancer and confer substantial progression risk factors.^17^ Moreover, variant histology tumors are more often diagnosed at an advanced stage.^17^ Recent multicenter reports suggest that immediate cystectomy should be preferred in the case of T1HG with pure squamous or micropapillary differentiation.^18^ Multiple tumor locations and large size are regarded as risk factors for recurrence and progression according to several available nomograms.^1–3,19^ Our data demonstrated that the location of the tumor also matters—multiple site lesions and tumors of overlapping sites have worse prognoses. The prognostic value of tumor location has not been well evidenced yet. Recently, Fukushima et al. showed that among intermediate-risk tumors, bladder neck involvement sub-stratifies patients according to the risk of progression.^20^

Sociodemographic factors such as race, income, and marital status are also associated with CSS, but their relevance is less pronounced than that of clinical and histopathological tumor features and patient’s age. Conversely, another population-based study showed that OS and RFS do not differ between White and Black patients in the setting of equal access to healthcare resources.^21^ Income and financial status undoubtedly affect access to novel therapies and perhaps to high-quality healthcare. Marital status underlines the significance of relatives’ support in the patient’s adherence to the urologist’s recommendations and struggle against cancer. A study by Heyes et al.^22^ demonstrated the value of a supportive partner in dealing with the effects of bladder cancer daily and underlined the importance of partner adaptation to their disease, surveillance, and treatment protocols.

Prediction of CSS, which is the most significant ultimate end-point in oncological studies, is of high relevance in NMIBC in order to identify the group of highest-risk patients, who should be qualified for intensified treatment and very stringent follow-up. A novel therapeutic scheme is still awaited for high-risk NMIBC. An aggressive therapeutic approach might include RC or, in the near future, a combined adjuvant therapy after complete TURBT accompanied by intensive cystoscopic surveillance.^23^ Awaited results of ongoing trials with a BCG and immune checkpoint inhibitors combination might soon change the current gold standard therapy for high- and very-high-risk subgroups, making risk-stratification even more important.

Despite its strengths, our study has certain limitations which must be acknowledged. T1HG diagnosis might have potentially included a proportion of understaged tumors (we lacked data about detrusor muscle in the specimen and about repeated TURBT performance). Data about tumor multiplicity was not precise and based solely on ICD-10 C67 coding. Primary tumor status was defined accordingly, with the SEER coding manual, and the cohort included only 8% of patients with recurrent tumors. The data about comorbidities and smoking status which constitute confounders in the survival analysis were not available.^24^ We did not analyze the subsequent therapies after TURBT and the data about recurrence and progression were not available. On the other hand, we managed to analyze a very large cohort of patients with NMIBC showing real-world long-term survival outcomes. Although this population-based study has several unavoidable limitations, we believe that it provides important CSM data and a clinically useful nomogram.

## Conclusions

To conclude, NMIBC is associated with a significant risk of long-term cancer-specific mortality especially, but not only, in patients with T1HG tumors. Relatively rarely diagnosed TaHG and T1LG tumors require attention as they are associated with approximately 10% CSM and therefore should be regarded as high-risk. T stage, grade, and patient’s age remain the most powerful determinants of CSS, but sociodemographic factors might also impair the prognosis. Further studies aiming for better stratification of risk in NMIBC are warranted.

### Supplementary Information

Below is the link to the electronic supplementary material.

Supplementary file1 (DOCX 18 KB)

## Data Availability

The datasets analyzed during the current study are available from the U.S. National Cancer Institute upon request.
