# Supplementary material for: Cancer-Specific Survival of Patients with Non-Muscle-Invasive Bladder Cancer: A Population-Based Analysis
Source: Ann Surg Oncol. 2023 Aug 14;30(12):7892–902. doi: 10.1245/s10434-023-14051-9 (PMC10562346; doi:10.1245/s10434-023-14051-9)
Supplement: Supplementary file 1 — Supplementary file1 (DOCX 18 KB) [file 10434_2023_14051_MOESM1_ESM.docx]

Supplementary file 1

**Supplementary Table.1. Multivariable analysis with Cox proportional hazards for the prediction of overall survival of patients with non-muscle-invasive bladder cancer.**

| Factors predicting overall survival- multivariable analysis | | | | |
| --- | --- | --- | --- | --- |
| Variables | | HR | 95% CI | P-value |
| Tumour T category and grading | TaLG | ref |  | <.0001 |
|  | T1LG | 1.266 | 1.204-1.331 | <.0001 |
|  | TaHG | 1.134 | 1.090-1.180 | <.0001 |
|  | Tis | 1.266 | 1.142-1.405 | <.0001 |
|  | T1HG | 1.497 | 1.448-1.548 | <.0001 |
| Age (years) | <60 | ref |  | <.0001 |
|  | 60-70 | 2.203 | 2.070-2.344 | <.0001 |
|  | 70-80 | 4.587 | 4.327-4.861 | <.0001 |
|  | >80 | 10.229 | 9.652-10.840 | <.0001 |
| Gender | male vs female | 1.303 | 1.260-1.349 | <.0001 |
| Tumour size | ≥ 3 cm vs < 3 cm | 1.120 | 1.089-1.152 | <.0001 |
| Tumour histology | urothelial | ref |  | <.0001 |
|  | squamous | 1.299 | 1.114-1.516 | 0.0009 |
|  | other variants | 1.292 | 1.080-1.545 | 0.0050 |
| Tumour location | lateral/posterior/trigone | ref |  | <.0001 |
|  | anterior/dome | 1.059 | 1.001-1.120 | 0.0462 |
|  | bladder neck | 0.993 | 0.913-1.080 | 0.8727 |
|  | more than one area | 1.119 | 1.068-1.171 | <.0001 |
|  | not specified/multiple | 1.058 | 1.025-1.092 | 0.0005 |
| History of previous NMIBC | yes vs no | 1.496 | 1.318-1.699 | <.0001 |
| Marital status | married* | ref |  | <.0001 |
|  | unmarried | 1.355 | 1.315-1.396 | <.0001 |
|  | status unkown | 1.107 | 1.032-1.188 | 0.0048 |
| Race | white | ref |  | <.0001 |
|  | black | 1.078 | 1.007-1.155 | 0.0310 |
|  | other** | 0.742 | 0.692-0.795 | <.0001 |
| Income annually | < $65,000 vs ≥ $65,000 | 1.114 | 1.081-1.148 | <.0001 |
| Metropolitan citizenship*** | yes vs no | 0.902 | 0.866-0.939 | <.0001 |

* partnership without official marriage was also regarded as married status

**includes American Indian/Alaska Native/Asian or Pacific Islander

*** citizenship of counties in a metropolitan area
